# The Effect of Aversive Therapy Using Virtual Reality on Craving, Depression, and Self‐Efficacy: A Pilot Study in Patients Under Methadone Maintenance Treatment

**DOI:** 10.1002/brb3.70953

**Published:** 2025-10-11

**Authors:** Nasim Sadeghi, Elham Sadat Mousavinasab, Ehsan Sharifi, Fatemeh Sadat Ghoreishi, Amir Ghaderi, Abdollah Omidi

**Affiliations:** ^1^ Department of Addiction Studies, School of Medicine Kashan University of Medical Sciences Kashan Iran; ^2^ Clinical Research Development Unit‐Matini/Kargarnejad Hospital Kashan University of Medical Sciences Kashan Iran; ^3^ Virtual Education Center Kashan University of Medical Sciences Kashan Iran; ^4^ Research and Planning Expert of Presidential Anti‐Narcotics Headquarters Isfahan Iran; ^5^ Department of Clinical Psychology, School of Medicine Kashan University of Medical Sciences Kashan Iran

**Keywords:** aversion therapy, depression, methadone maintenance therapy, self‐efficacy, substance craving, virtual reality

## Abstract

**Objective:**

This study aims to evaluate the effectiveness of virtual reality‐based aversion therapy in reducing craving, alleviating depression, and improving self‐efficacy in patients with substance use disorders undergoing methadone maintenance therapy.

**Methods:**

The research was conducted as an interventional clinical trial involving 90 participants aged 25 to 55 years from outpatient treatment centers. The participants were randomly assigned to one of three groups: a group that received virtual reality‐based aversion therapy combined with methadone, a group that received psychological counseling combined with methadone, and a group that received methadone only.

**Results:**

The findings revealed significant differences between the groups' means for depression (*F* = 19.652, *p* = 0.000), self‐efficacy (*F* = 33.956, *p* = 0.000), and craving (*F* = 65.445, *p* = 0.000 for desire and intent to use substances; *F* = 45.931, *p* = 0.000 for craving and negative reinforcement; *F* = 76.202, *p* = 0.000 for pleasure and intensity of lack of control). Specifically, virtual reality‐based aversion therapy significantly reduced the desire and intent to use substances, craving and negative reinforcement, and pleasure and intensity of lack of control compared to both psychological counseling and control groups. It also significantly reduced depression and enhanced self‐efficacy compared to both psychological counseling and control groups. This therapy, implemented through Python programming in a virtual reality environment with interactive and controlled simulations, facilitated gradual exposure to negative stimuli, cognitive restructuring, and the reinforcement of positive behaviors.

**Conclusion:**

The results underscore the high potential of virtual reality–based aversion therapy to improve the effectiveness and quality of substance abuse treatment. This study emphasizes the necessity of further research in this field to improve therapeutic interventions and presents virtual reality‐based aversion therapy as an innovative, complementary, or alternative approach to substance use treatment.

## Graphical abstract

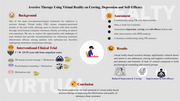

## 1 Introduction

Methadone is one of the therapeutic approaches for treating drug addiction, commonly used as maintenance therapy for patients with a history of substance use. In this approach, the medication dose is gradually increased until withdrawal symptoms subside, and cravings for illicit substances are minimized (Joseph et al. 2000). Methadone maintenance therapy usually lasts for an extended period, at least several months, often exceeding a year, and in some cases, continues for a lifetime (Joseph et al. 2000). This treatment not only prevents relapse into substance use but also improves the physical and mental health of patients (Reichert et al. 2025; Verdejo‐García et al. 2004).

In methadone maintenance therapy, the initial dosage usually starts at 30 mg and is adjusted to reach an optimal dose. After dose stabilization, patients are evaluated for other chronic medical and psychiatric disorders (Heimer et al. 2002). However, patients undergoing methadone therapy often exhibit poor performance in areas such as processing speed, visuospatial attention, and cognitive flexibility tests (Reichert et al. 2025). Additionally, the Centers for Disease Control and Prevention (CDC) has raised concerns regarding opioid dependence and the rising costs associated with substance use and similar medications in patients receiving primary care (Cipresso et al. 2018). The CDC emphasizes the need to develop therapeutic methods, including innovative non‐pharmacological approaches, to reduce opioid overuse and prevent associated complications (Cipresso et al. 2018).

Aversion therapy, based on classical conditioning theory, is one of the main non‐pharmacological treatments for addiction. Aversion therapy aims to reduce or eliminate undesirable behaviors by conditioning individuals to associate those behaviors with an unpleasant stimulus (Jaqua and Jaqua 2019). Aversion therapy utilizes electrical, chemical, or imagery stimuli (Jaqua and Jaqua 2019). The use of virtual reality (VR) as a tool for delivering these stimuli has proven highly effective. In conjunction with aversion therapy, VR not only fosters a sense of mastery and efficacy in the individual but also enables precise control of stimuli for the therapist, making it an efficient method for treating various addictions (Verdejo et al. 2005).

VR creates computer‐generated models of the real world, allowing users to interact with these models through novel human–computer interfaces, thereby providing an almost real experience (Roelofsen and Carter‐White 2022). In recent years, VR hardware and software have been increasingly used across commercial, educational, clinical, and research domains (Blach 2008). VR is widely employed for diagnosing, assessing, and treating mental disorders (Freitas and Spadoni 2019; Ferreri et al. 2018).

Overall, the application of VR has been shown to improve mood, enhance perceived social support, increase satisfaction and participation in treatment (Mahapatra 2021; Voinescu et al. 2023), reduce cravings (Tsai et al. 2021), decrease substance use and substance‐related urges (Taubin et al. 2023), improve self‐efficacy, and lower anxiety and depression (Lin and Wang 2021; Zheng et al. 2021; Shahrbaf et al. 2024). In the future, VR could bring transformative advancements to addiction treatment by offering novel therapeutic models with significant benefits for assessing and treating addictive behaviors while achieving a high level of ecological validity (Segawa et al. 2020; Langener et al. 2021).

Therefore, the primary objective of this study is to evaluate the effectiveness of VR‐based aversion therapy compared to conventional treatment methods, including counseling and methadone maintenance therapy, in addressing craving, depression, and self‐efficacy among individuals with substance‐use disorders. This study aims to explore the opportunities and challenges of each method and provide recommendations for enhancing treatment effectiveness. By leveraging VR's ability to create realistic and controlled simulated environments, aversion therapy could not only reduce cravings and improve patients' mental health but also serve as an innovative and complementary approach to enhancing treatment quality and increasing patient engagement in the recovery process.

## 2 Materials and Methods

### 2.1 Ethical Considerations

The methodology of this study adhered to international regulations and was approved by the Ethics Committee of Kashan University of Medical Sciences (Ethics Code^1^: IR.KAUMS.MEDNT.REC.1403.007). Before participating, all individuals were informed about the study process, and written consent was obtained under the supervision of an addiction specialist. The study incurred no costs for the participants and was funded through a specialized project by an international campus student, Dr. Nasim Sadeghi. Participation was entirely voluntary, and patients provided informed written consent with the assurance of confidentiality (Clinical Trial Registration Code: IRCT20240429061588N1). Participants were allowed to withdraw from the study at any stage upon request.

### 2.2 Study Design

This interventional clinical trial was conducted on 90 individuals aged 25–55 undergoing methadone maintenance therapy (MMT) in Tehran and Kashan (methadone addiction treatment center under the supervision of the vice president of Kashan University). It represents the first application of VR‐based aversion therapy for substance‐use disorder treatment in Iran, aiming to introduce it as a supportive tool for addiction therapy. Participants were selected from outpatient treatment centers in Kashan and Tehran, operating under the supervision of the vice‐chancellor for treatment in the years 2023 and 2024. Inclusion criteria required a minimum of 3 months of methadone use without relapse.

### 2.3 Participants and Sampling Method

This pilot study was conducted on individuals who had been undergoing methadone maintenance therapy (MMT) at government‐supervised centers in Kashan and Tehran for at least 3 months without relapse into substance use. Eligible participants were selected based on predefined criteria. Initial sampling was conducted using a purposeful convenience method from individuals on stable‐dose methadone in the maintenance phase. Participants were then randomly assigned to one of the following three groups:

1. The VR‐based aversion therapy intervention group combined with methadone,
2. The psychological counseling group combined with methadone,
3. The methadone‐only group.

Random group allocation was performed using the Random Allocation Software. Given the pilot nature of this study and the lack of similar research in Iran, the primary aim was to prevent resource wastage in the future with more extensive studies. The pilot study allowed for identifying and addressing potential implementation challenges, enabling a more accurate design for subsequent research. Regarding the craving rate as the main outcome, no study was found to calculate the sample size in patients under methadone maintenance treatment. However, considering the possibility of sample loss and to enhance reliability and generalizability, this study selected 90 methadone‐maintained patients in three groups. After compiling a list of eligible patients, participants meeting the inclusion criteria were randomly distributed into three groups of 30. The substance use history of the 90 participants varied considerably, ranging from daily opium and heroin use to cases like a patient on tramadol who had been mistakenly started on methadone by another physician. All participants met the DSM‐5 criteria for substance use disorder and were stabilized in the methadone maintenance phase at the time of recruitment, with no relapses for the past 3 months. The number of previous treatment attempts varied, from first‐time treatment to a maximum of four attempts (including treatment failures). This number, however, was not an inclusion criterion; the key requirements were current maintenance treatment and stability.

Inclusion Criteria: Participants had to visit the clinic at least once a week, be aged between 25 and 55, and have been on a stable methadone dose for 3 months or more. They were required to demonstrate willingness to participate by signing an informed consent form. Other inclusion criteria included basic literacy, the ability to communicate verbally, no severe vision or hearing impairments, and no history of frequent or severe headaches. Participants with prior psychiatric disorders, epilepsy, malignancies, or signs of stimulant or opioid abuse were excluded, as were those receiving psychiatric treatment for cognitive or psychosomatic disorders. As per the inclusion criteria, the sole permitted medication at study baseline was methadone maintenance therapy; use of all other medications, especially psychiatric drugs, was an exclusion criterion.

Exclusion Criteria: Participants could withdraw at any stage if they chose to discontinue their involvement. Additionally, urine tests for morphine, amphetamines, and cannabis were administered three times during the study. A positive test result disqualified the individual from further participation. Other exclusion criteria included the onset of psychotic symptoms, pregnancy in female participants, and the use of psychiatric or neurological medications affecting cognition, such as antipsychotics.

### 2.4 Instruments and Data Collection Methods

Before entering the study, all participants were examined by a general practitioner, and demographic questionnaires capturing age, gender, education, marital status, occupation, and duration of methadone use were completed. For a more detailed assessment, three standardized tools were administered at two time points (before the study and after 12 weeks):

1. *Desire for Drug Questionnaire (DDQ)*: Developed by Franken et al. 2002., this instrument evaluates current drug cravings across three dimensions: “desire and intention to use substances,” “urge to use and negative reinforcement,” and “pleasure and intensity of loss of control.” The questionnaire contains 14 items rated on a 6‐point Likert scale ranging from “*not true at all*” (0) to “*completely true*” (5). Its reliability and validity have been confirmed with Cronbach's alpha ranging from 0.75 to 0.89 (Langener et al. 2021).
2. *Bramson's Addiction Self‐Efficacy Questionnaire*: Designed by Bramson (1999), this 16‐item questionnaire assesses skills in problem‐solving, decision‐making, assertiveness, and communication to measure addiction self‐efficacy. Responses are scored on a 7‐point Likert scale (from “*definitely no*” to “*definitely yes*”), with scores ranging from 16 to 112. The tool demonstrates high reliability, with a Cronbach's alpha of 0.90, and satisfactory validity based on factor analysis (Habibi et al. 2012).
3. *Beck Depression Inventory (BDI)*: This 21‐item questionnaire, created by Beck et al. (1961), evaluates depressive symptoms with a focus on cognitive content. Responses are rated on a 4‐point Likert scale, with higher scores indicating greater depression severity. The BDI has demonstrated reliability with a Cronbach's alpha of 0.87 and validity supported by numerous domestic and international studies.

Participants underwent free weekly urine drug tests, and those with positive results were excluded from the study. During the study, three patients were excluded due to positive urine tests for morphine and amphetamines and unwillingness to continue participation. These individuals were replaced with three other eligible volunteers.

### 2.5 Intervention Method

This study lasted 12 weeks, during which participants were instructed to maintain their normal physical activity and dietary routines. Patients were informed that their required methadone would be provided free of charge during this period to encourage regular attendance at sessions and compliance with addiction tests.

**FIGURE 1:**
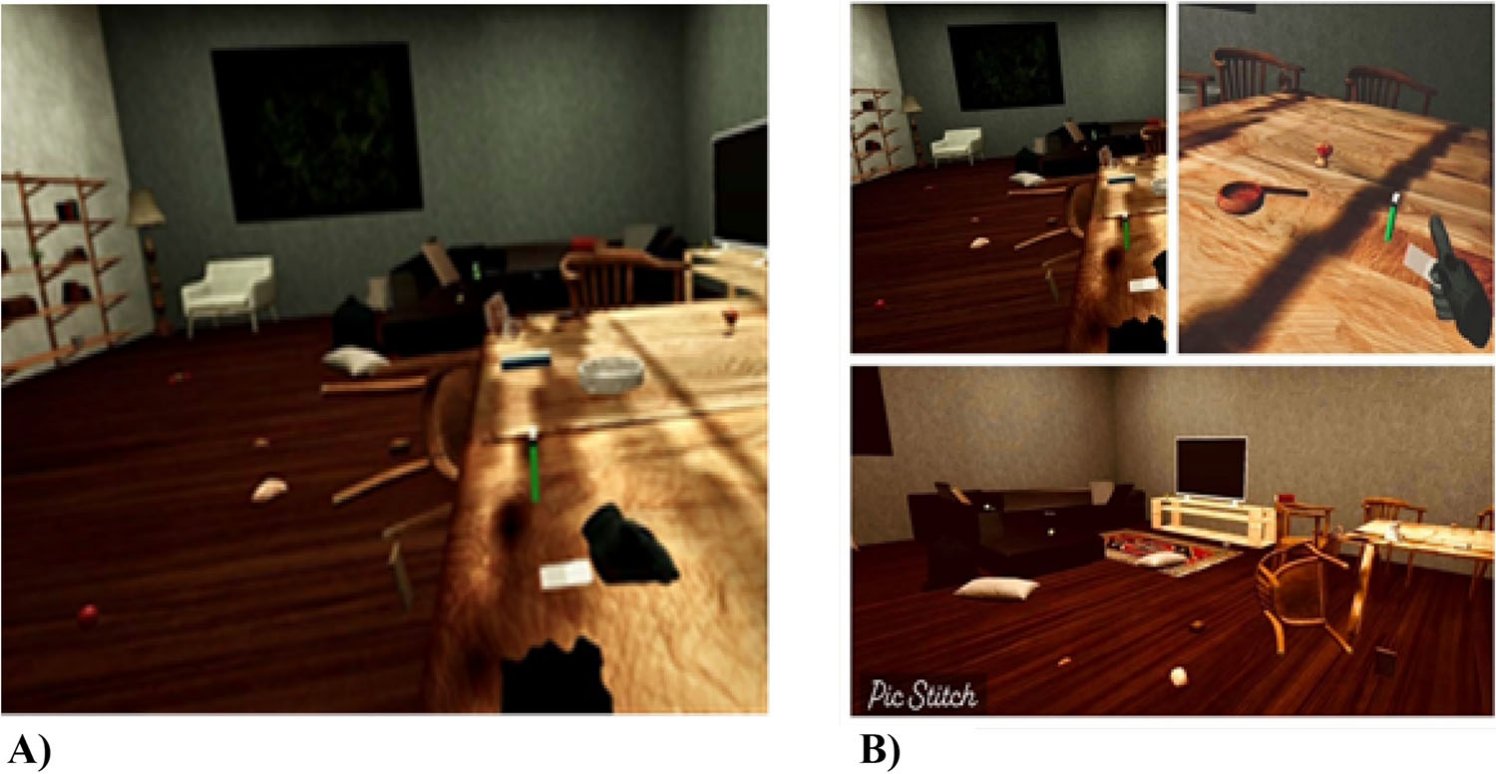
Two screenshots of some of the VR images.

1. *VR‐Based Intervention*: The VR software was developed by computer science and IT experts at Pioneers of Iranian VR Company. The software simulates a virtual environment containing stimuli that either trigger drug cravings or elicit aversive responses. Users actively interact in this space, practicing positive behaviors such as abstinence, destroying drug paraphernalia, and organizing and cleaning their surroundings. The scenario was designed in such a way that participants entered a messy room. Books, pillows, and rotten fruit were scattered around. Participants were asked to tidy the room. He could arrange the shelves and the items. By tidying the room, he would earn positive points. Suddenly, he would see drug paraphernalia on the table. By touching the drug paraphernalia, an annoying and unpleasant sound would be played through the headset, resulting in a negative experience and the assignment of penalty points. To complete the game, the participant had to touch the drug paraphernalia, and this interaction had to occur in every session. This intervention takes place once a week and lasts about 6 min and ends with touching the drug paraphernalia to achieve an aversion during each session. In subsequent sessions, participants are more likely to choose avoidance behaviors to avoid negative consequences and earn higher scores. To enhance motivation, calming and encouraging sounds are played, and positive behaviors are rewarded with bonus points. Immersion in the program's three‐dimensional virtual environment is time‐consuming. Therefore, two screenshots from the VR scenario are provided (Figure 1).
2. *Psychological Counseling Group*: Counseling sessions in this group are conducted by the center's psychologist, who holds a master's degree in psychology and has received specialized training in the Methadone maintenance treatment protocol of the Iranian Ministry of Health (MMT) and Cognitive Behavioral Therapy (CBT). The psychologist can either continue sessions based on patients' individual needs or start afresh if required. Given that patients have already been treated, the treatment process, which in Iran is mainly CBT, will continue. The content of the counseling sessions will start and continue depending on the diagnosis of the disorder and the needs of the participant.
3. *Methadone‐Only Group*: This group solely receives methadone without any additional interventions.

### 2.6 Statistical Analysis

Statistical analysis was performed using SPSS software version 30. Descriptive statistics were initially used to summarize the data. Given that the assumptions for parametric statistical tests were not met, nonparametric Wilcoxon tests were employed to assess significant pre‐ and post‐intervention changes within groups. Additionally, nonparametric analysis of covariance was used to evaluate significant differences between groups, with 95% confidence intervals calculated to estimate the precision of the group effects.

## 3 Results

**FIGURE 2:**
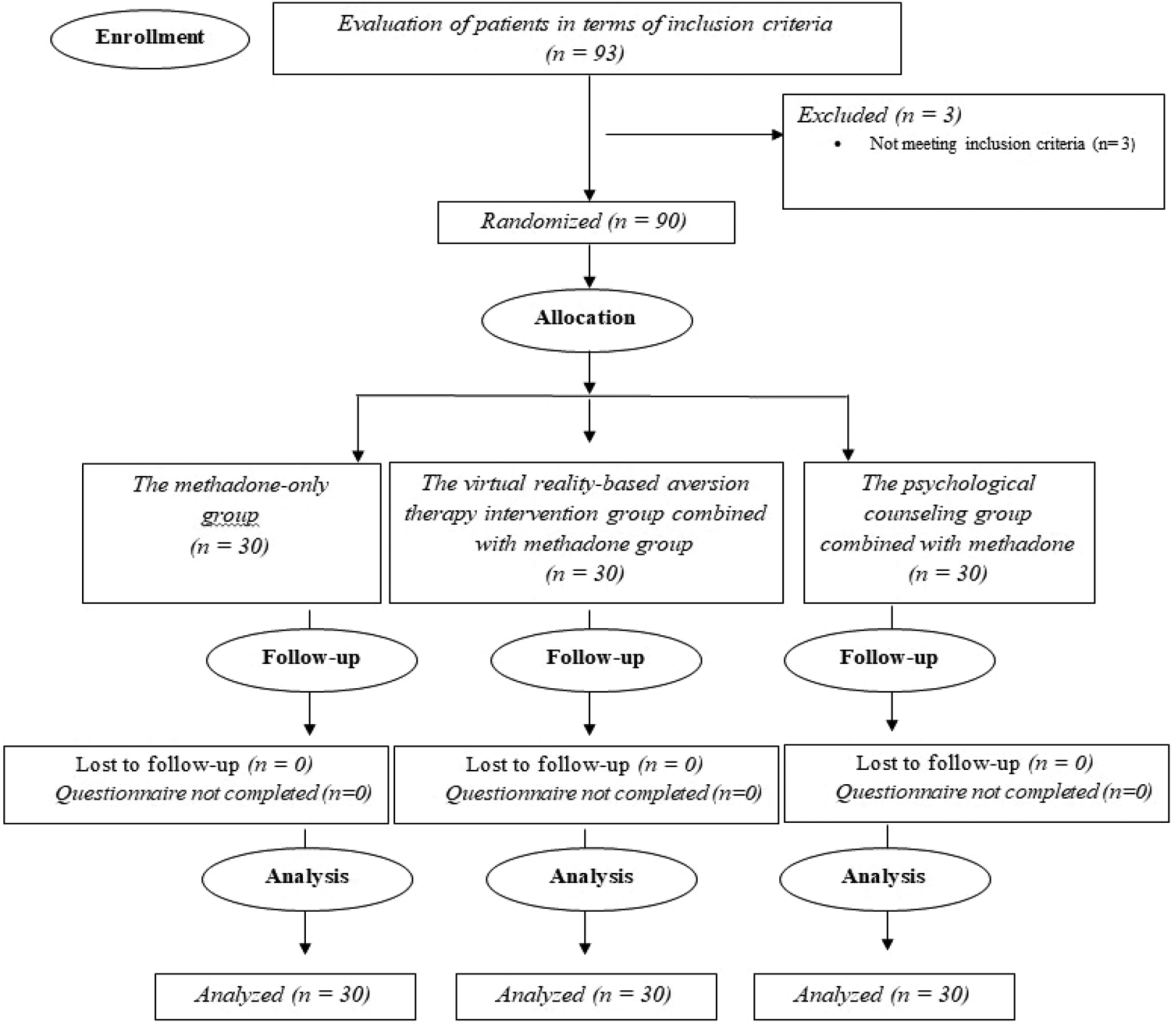
Flowchart of the clinical trial.

Out of 93 screened participants, 90 patients were randomized into the methadone‐only group (*n* = 30), the VR‐based aversion therapy intervention group combined with methadone group (*n* = 30), and the psychological counseling group combined with methadone (*n* = 30) (Figure 2). None of the patients dropped out of the study because the questionnaire was not completed or due to side effects. Figure 2 indicates the flow diagram of CONSORT regarding patient registration.

The demographic information of the patient is presented in Table 1. Based on the data in Table 1, the average age in the VR‐based aversion therapy group is 44.04 years, in the psychological counseling group is 43.53 years, and in the control group is 41.40 years. The VR‐based aversion therapy group has the longest history of drug use, with an average of 5.36 years, compared to 4.02 years in the psychological counseling group and 4.08 years in the control group. The average age of first substance use in the VR‐based aversion therapy group is 21.29 years, in the psychological counseling group it is 19.68 years, and in the control group it is 24.67 years. Lastly, the mean daily dose of methadone is 70.88 mg in the VR‐based aversion therapy group, 40.75 mg in the psychological counseling group, and 72.90 mg in the control group. Specifically, participants were on a maintenance phase of methadone treatment.

**TABLE 1:** Demographic information of patients.

Variables		VR‐based aversion therapy	Psychological counseling	Control
Gender (*n*)	Male	28	28	29
	Female	2	2	1
Age	Mean	44.04	43.53	41.4
	Std. deviation	7.73	5.76	6.45
History of drug use (year)	Mean	5.36	4.02	4.08
	Std. deviation	3.47	2.34	3.66
Age of first substance use	Mean	21.29	19.68	24.67
	Std. deviation	9.29	8.48	5.67
Daily dose of methadone (mg)	Mean	70.88	40.75	72.9
	Std. Deviation	40.64	33.13	20.73

Based on the data in Table 2, the mean depression scores in the VR‐based aversion therapy group combined with methadone significantly decreased from 15.533 to 13.267 (*Z* = −2.602, *p* = 0.009). Similarly, the desire and intent to use substances dropped significantly from 9.1 to 4.433 (*Z* = −4.791, *p* = 0.000). Craving and negative reinforcement decreased significantly from 9.167 to 4.467 (*Z* = −4.223, *p* = 0.000). Pleasure and intensity of lack of control were reduced significantly from 11.3 to 5.367 (*Z* = −4.725, *p* = 0.000). Furthermore, self‐efficacy showed a significant increase from 64.367 to 78.233 (*Z* = −4.309, *p* = 0.000).

**TABLE 2:** Evaluation of study variables pre‐ and post‐intervention.

	Time	Mean	Std. Deviation	*p* value	*Z*
VR‐based aversion therapy					
Depression	Pre	15.533	6.932	0.009	−2.602
	Post	13.267	6.034		
Desire and intent to use substances	Pre	9.100	4.686	0.000	−4.791
	Post	4.433	2.555		
Craving and negative reinforcement	Pre	9.167	4.316	0.000	−4.223
	Post	4.467	2.874		
Pleasure and intensity of lack of control	Pre	11.300	5.364	0.000	−4.725
	Post	5.367	2.697		
Self‐efficacy	Pre	64.367	9.543	0.000	−4.309
	Post	78.233	10.143		
Psychological counseling					
Depression	Pre	14.000	6.818	0.000	−3.922
	Post	9.800	4.902		
Desire and intent to use substances	Pre	8.633	4.335	0.001	−3.185
	Post	7.133	3.589		
Craving and negative reinforcement	Pre	8.433	4.108	0.040	−2.051
	Post	7.500	3.839		
Pleasure and intensity of lack of control	Pre	10.600	5.295	0.020	−2.331
	Post	9.0670	4.518		
Self‐efficacy	Pre	61.367	12.455	0.948	−0.065
	Post	61.067	12.06		
Control					
Depression	Pre	14.300	7.957	0.002	−3.105
	Post	15.667	8.036		
Desire and intent to use substances	Pre	8.467	3.812	0.000	−4.247
	Post	10.300	4.053		
Craving and negative reinforcement	Pre	10.200	3.800	0.090	−1.696
	Post	10.633	3.774		
Pleasure and intensity of lack of control	Pre	12.633	4.575	0.044	−2.010
	Post	13.333	4.381		
Self‐efficacy	Pre	74.067	8.081	0.000	−3.931
	Post	68.600	10.112		

In the psychological counseling group combined with methadone, the mean depression scores significantly decreased from 14.000 to 9.800 (*Z* = −3.922, *p* = 0.000). The desire and intent to use substances also showed a significant decrease from 8.633 to 7.133 (*Z* = −3.185, *p* = 0.001). Craving and negative reinforcement decreased significantly from 8.433 to 7.500 (*Z* = −2.051, *p* = 0.040). Pleasure and intensity of lack of control decreased significantly from 10.600 to 9.067 (*Z* = −2.331, *p* = 0.020). Self‐efficacy showed no significant change, slightly decreasing from 61.367 to 61.067 (*Z* = −0.065, *p* = 0.948).

In the methadone‐only group, the mean depression scores significantly increased from 14.3 to 15.667 (*Z* = −3.105, *p* = 0.002). The desire and intent to use substances also showed a significant increase from 8.467 to 10.3 (*Z* = −4.247, *p* = 0.000). Craving and negative reinforcement did not show a significant change, increasing slightly from 10.2 to 10.633 (*Z* = −1.696, *p* = 0.090). Pleasure and intensity of lack of control increased slightly but significantly from 12.633 to 13.333 (*Z* = −2.010, *p* = 0.044). Self‐efficacy significantly decreased from 74.067 to 68.6 (*Z* = −3.931, *p* = 0.000).

The VR‐based aversion therapy group combined with methadone demonstrated the most significant reductions in depression, desire and intent to use substances, craving and negative reinforcement, and pleasure and intensity of lack of control. Additionally, this group experienced the most significant increase in self‐efficacy. The psychological counseling group combined with methadone also showed reductions in these variables, but the changes were less pronounced, and self‐efficacy showed no meaningful change. Conversely, the methadone‐only group not only failed to achieve significant reductions in negative variables but also experienced significant increases in depression and the desire and intent to use substances.

To further evaluate the effectiveness of VR‐based aversion therapy compared to psychological counseling and methadone maintenance therapy, the effects of the group on craving, depression, and self‐efficacy were assessed, while controlling for time, using nonparametric analysis of covariance. The results are detailed in Table 3.

**TABLE 3:** Evaluation of the comparison of groups in the posttest.

	*F*	*p* value	Mean difference	*p* value
Depression				
VR vs. PC	19.652	0.000	2.318	0.037
VR vs. Ctrl			−3.324	0.000
PC vs. Ctrl			−5.642	0.000
Desire and intent to use substances				
VR vs. PC	65.445	0.000	−2.998	0.000
VR vs. Ctrl			−6.271	0.000
PC vs. Ctrl			−3.273	0.000
Craving and negative reinforcement				
VR vs. PC	45.931	0.000	−3.525	0.000
VR vs. Ctrl			−5.474	0.000
PC vs. Ctrl			−1.95	0.004
Pleasure and intensity of lack of control				
VR vs. PC	76.202	0.000	−4.15	0.000
VR vs. Ctrl			−7.11	0.000
PC vs. Ctrl			4.15	0.000
Self‐efficacy				
VR vs. PC	33.956	0.000	15.114	0.000
VR vs. Ctrl			16.172	0.000
PC vs. Ctrl			1.027	1.000

Based on the data in Table 3, for depression, a significant difference was observed between the groups' means (*F* = 19.652, *p* = 0.000). Specifically, VR‐based aversion therapy, compared to the psychological counseling group combined with methadone, resulted in a significant reduction in depression (mean difference = 2.318, *p* = 0.037). Additionally, the comparison between the VR‐based aversion therapy group and the control group showed that this method was significantly more effective in reducing depression (mean difference = −3.324, *p* = 0.000). Furthermore, the psychological counseling group also demonstrated a significant reduction in depression compared to the control group (mean difference = −5.642, *p* = 0.000). These results highlight the positive impact of both treatment methods, with VR‐based aversion therapy being more effective.

For desire and intent to use substances, a significant difference was observed between the groups' means (*F* = 65.445, *p* = 0.000). VR‐based aversion therapy was significantly more effective in reducing cravings compared to the psychological counseling group (mean difference = −2.998, *p* = 0.000) and the control group (mean difference = −6.271, *p* = 0.000). The psychological counseling group also showed a notable reduction in cravings compared to the control group (mean difference = −3.273, *p* = 0.000).

For craving and negative reinforcement, a significant difference was observed between the groups' means (*F* = 45.931, *p* = 0.000). VR‐based aversion therapy outperformed both the psychological counseling group (mean difference = −3.525, *p* = 0.000) and the control group (mean difference = −5.474, *p* = 0.000). Additionally, a significant difference was observed between the psychological counseling group and the control group (mean difference = −1.95, *p* = 0.004).

For pleasure and intensity of lack of control, a significant difference was observed between the groups' means (*F* = 76.202, *p* = 0.000). VR‐based aversion therapy demonstrated significant effectiveness compared to the psychological counseling group (mean difference = −4.15, *p* = 0.000) and the control group (mean difference = −7.11, *p* = 0.000). Similarly, the psychological counseling group showed significantly better performance than the control group in this variable (mean difference = 4.15, *p* = 0.000).

For self‐efficacy, a significant difference was observed between the groups' means (*F* = 33.956, *p* = 0.000). The VR‐based aversion therapy group showed a significant increase compared to both the psychological counseling group (mean difference = 15.114, *p* = 0.000) and the control group (mean difference = 16.172, *p* = 0.000). However, no significant difference was observed between the psychological counseling group and the control group (mean difference = 1.027, *p* = 1.000). These findings underscore the remarkable impact of VR‐based aversion therapy on enhancing self‐efficacy.

## 4 Discussion

This study aimed to evaluate the effectiveness of VR‐based aversion therapy on craving, depression, and self‐efficacy in patients with substance‐use disorders undergoing methadone maintenance therapy (MMT). The findings indicate that VR‐based aversion therapy had significant positive effects in reducing depression and substance cravings and enhancing self‐efficacy. Furthermore, VR‐based aversion therapy was significantly more effective than psychological counseling combined with MMT or MMT alone.

The results demonstrated that the group that received VR‐based aversion therapy experienced the most significant reduction in depression. These findings align with studies by Voinescu et al. (2023) and Shahrbaf et al. (2024), which reported that VR can help alleviate symptoms of depression (Voinescu et al. 2023; Shahrbaf et al. 2024). The likely reason for this effectiveness lies in the unique features of VR, including creating immersive, multisensory experiences and personalized scenarios that allow patients to engage actively and positively with their emotions. By offering an alternative way of coping with negative feelings, VR may help patients break the cycle of negative thoughts and rumination associated with depression, steering their focus toward more adaptive strategies. While the psychological counseling group also showed a significant reduction in depression, the extent of the reduction was smaller compared to the VR‐based method. This highlights the potential advantage of interactive, multidimensional environments in VR over traditional therapeutic approaches. Conversely, the MMT‐only group exhibited a significant increase in depression, likely due to their inability to address the psychological and emotional aspects of substance use and its consequences. This underscores the limitations of MMT alone in reducing depression and highlights the need for complementary interventions.

In terms of substance cravings, the VR‐based aversion therapy group exhibited a notable and significant reduction in craving and intent to use, desire to consume, and negative reinforcement. These findings are consistent with the studies by Segawa et al. (2020) and Tsai et al. (2021), which demonstrated that VR can reduce substance cravings by providing safe and controlled stimuli (Segawa et al. 2020; Tsai et al. 2021). The efficacy of VR in this regard stems from its ability to recreate real‐life substance‐use scenarios and foster a sense of aversion towards these behaviors. This technique may recondition patients' responses to substance‐related cues, ultimately reducing cravings. Although the counseling group also experienced a reduction in substance cravings, the magnitude of this reduction was smaller compared to the VR group. This could be attributed to the limitations of traditional counseling in recreating real‐life experiences and addressing conditioned responses. Conversely, the MMT‐only group demonstrated a significant increase in cravings, likely due to the inability of methadone alone to manage the psychological and environmental triggers associated with substance use. These findings collectively underscore the significant potential of VR‐based aversion therapy as a powerful complementary tool for improving outcomes in individuals undergoing treatment for substance‐use disorders.

This study found that the VR‐based aversion therapy group experienced a significant increase in self‐efficacy, whereas no meaningful change was observed in the psychological counseling group, and a significant decrease was noted in the group that only consumed methadone. These results align with the findings of Zheng et al. (2021) and Daria Taubin et al. (2023), which highlighted the ability of VR to enhance patients' sense of empowerment and self‐efficacy through controlled and positive experiences. The improvement in self‐efficacy with VR therapy can be attributed to its interactive and dynamic nature, which allows patients to practice and strengthen their skills in simulated environments. This feature helps patients gain greater confidence in their ability to manage behaviors related to substance use. In contrast, the lack of significant improvement in self‐efficacy in the psychological counseling group and its decline in the methadone‐only group reflect the limitations of these approaches in fostering a sense of mastery and capability in patients.

Overall, the findings suggest that VR‐based aversion therapy is more effective than both psychological counseling with methadone and methadone‐only treatment in reducing depression, alleviating cravings, and enhancing self‐efficacy. This superiority can be attributed to the unique features of VR therapy, including its ability to simulate real‐life environments, control stimuli, and engage patients in active interactions. On the other hand, the weaker results observed in the methadone‐only group emphasize the necessity of incorporating complementary psychological interventions alongside pharmacological treatments.

The findings can be further explained by the unique features of VR‐based aversion therapy. These features include the ability to simulate realistic environments, create powerful sensory and emotional experiences, and engage patients actively. By modeling aversion to substances and modifying conditioned responses, this method reduces cravings and strengthens coping skills. Additionally, the interactive and dynamic nature of VR contributes to enhancing patients’ sense of control and self‐efficacy. In contrast, the limitations of traditional approaches, such as psychological counseling, in replicating real‐life scenarios, and the inability of methadone‐only treatment to address the psychological and emotional aspects of substance use, reduce their effectiveness and highlight the advantages of VR therapy.

Using innovative methods, such as VR, alongside traditional addiction treatment approaches can significantly improve recovery processes. This method, which was first applied in Iran, has the potential to pave the way for the integration of VR into substance abuse treatment. Convincing individuals with substance‐use disorders to attend weekly sessions was challenging and was only made possible by offering incentives, such as free methadone. Promoting a culture of VR‐assisted treatment is essential in the country. As VR‐based aversion therapy for addiction was implemented for the first time in Iran, it has laid the groundwork for developing more advanced software while addressing current limitations. Another major challenge was the lack of experience in designing VR scenarios for addiction treatment.

It is recommended that VR‐based treatment programs be designed with a long‐term, person‐centered approach to enable more sustainable outcomes. Additionally, developing content tailored to the specific needs of different groups, such as adolescents, individuals with unique disorders, and high‐risk populations, could enhance the effectiveness of this technology. Future research should focus on investigating the underlying mechanisms of VR's effectiveness, such as its impact on neuropsychological changes, and compare it to traditional treatment methods. Moreover, policymakers in the health and education sectors are encouraged to invest in this technology to promote its adoption in treatment and educational centers. In our study, the VR system successfully created a sense of presence through immersive three‐dimensional environments. However, implementing *embodiment* for all 90 participants would have required complex programming and individualized calibration, which was beyond the scope of the current project. We acknowledge this as a limitation of our study and suggest that future research should incorporate embodiment to further enhance the therapeutic potential of VR‐based aversion therapy. Although the programming costs of VR systems are high, we recommend that future studies develop devices capable of integrating olfactory stimuli into aversion therapy. This is because the human limbic system is evolutionarily more ancient and enduring in its processing of olfactory cues, which may enhance the effectiveness and durability of therapeutic outcomes.

## 5 Conclusion

This study demonstrated that VR‐based aversion therapy combined with methadone treatment significantly reduced cravings, depression, and the desire to use substances while enhancing self‐efficacy. Due to its realistic simulations and reinforcement of aversive responses, this method was more effective than counseling or methadone maintenance therapy alone. These findings underscore the substantial potential of VR as an innovative, effective method for alleviating psychological symptoms and enhancing recovery stability in individuals with substance use disorders.

## Author Contributions

All authors read and approved the final manuscript. All authors take responsibility for the integrity of the data and the accuracy of the data analysis.

## Ethics Statement

The patients were educated about the purpose of the study, and all patients gave their signed written informed consent letters. Ethical considerations were approved by the Kashan University of Medical Sciences research committee following the Declaration of Helsinki, with the ethical code: IR.KAUMS.MEDNT.REC.1403.007 (Clinical Trial Registration Code: IRCT20240429061588N1).

## Conflicts of Interest

The authors declare no conflicts of interest.

## Peer Review

The peer review history for this article is available at https://publons.com/publon/10.1002/brb3.70953.

## Data Availability

Data will be made available on request.

## Endnotes

1
https://ethics.research.ac.ir/PortalProposalList.php?code=IR.KAUMS.MEDNT.REC.1403.007&title=&name=&stat=&isAll=
